# Measurement of Scapholunate Joint Space Width on Real-Time MRI—A Feasibility Study

**DOI:** 10.3390/diagnostics14111177

**Published:** 2024-06-03

**Authors:** Jonathan Ehmig, Kijanosh Lehmann, Günther Engel, Fabian Kück, Joachim Lotz, Sebastian Aeffner, Ali Seif Amir Hosseini, Arndt F. Schilling, Babak Panahi

**Affiliations:** 1 Institute of Diagnostic and Interventional Radiology, University Medical Center Göttingen, 37075 Göttingen, Germany; 2 Department of Medical Statistics, University Medical Center Göttingen, 37073 Göttingen, Germany; 3 Clinic of Trauma, Orthopedics and Reconstructive Surgery, University Medical Center Göttingen, 37075 Göttingen, Germany

**Keywords:** scapholunate joint, real time MRI, FLASH, carpal instability, dynamic imaging

## Abstract

Introduction: The scapholunate interosseous ligament is pivotal for wrist stability, and its impairment can result in instability and joint degeneration. This study explores the application of real-time MRI for dynamic assessment of the scapholunate joint during wrist motion with the objective of determining its diagnostic value in efficacy in contrast to static imaging modalities. Materials and Methods: Ten healthy participants underwent real-time MRI scans during wrist ab/adduction and fist-clenching maneuvers. Measurements were obtained at proximal, medial, and distal landmarks on both dynamic and static images with statistical analyses conducted to evaluate the reliability of measurements at each landmark and the concordance between dynamic measurements and established static images. Additionally, inter- and intraobserver variabilities were evaluated. Results: Measurements of the medial landmarks demonstrated the closest agreement with static images and exhibited the least scatter. Distal landmark measurements showed a similar level of agreement but with increased scatter. Proximal landmark measurements displayed substantial deviation, which was accompanied by an even greater degree of scatter. Although no significant differences were observed between the ab/adduction and fist-clenching maneuvers, both inter- and intraobserver variabilities were significant across all measurements. Conclusions: This study highlights the potential of real-time MRI in the dynamic assessment of the scapholunate joint particularly at the medial landmark. Despite promising results, challenges such as measurement variability need to be addressed. Standardization and integration with advanced image processing methods could significantly enhance the accuracy and reliability of real-time MRI, paving the way for its clinical implementation in dynamic wrist imaging studies.

## 1. Introduction

The scapholunate interosseous ligament (SLIL) plays a crucial role in maintaining the stability and flexibility of the wrist during motion by connecting the scaphoid and lunate bones, and it also contributes to load transmission between the two carpal bones [1,2]. In healthy individuals, the ligament comprises three portions: a thicker dorsal portion, a fibrocartilagenous and biomechanically less important proximal segment and a thinner volar portion. Together, these form a C-shape, leaving the distal bone poles of the scaphoid and lunate bones unattached [3]. Biomechanical studies on ligament stiffness have yielded inconclusive results regarding regional differences within the SLIL [4,5]. The radius and the carpal bones are connected by a number of ligaments, which act as secondary stabilizers [2]. The radiocarpal and midcarpal joints function as a unit with a wide range of wrist motion and complex kinematics. When focusing on the scapholunate joint (SLJ), flexion and radial deviation result in a palmar inclination of the distal scaphoid, causing the lunate to be pulled in the same direction due to the strong SLIL. On the other hand, ulnar deviation and wrist extension lead to a dorsal incline of the scaphoid and lunate [2,6,7].

Damage to the SLIL can result in wrist instability, leading to altered biomechanics and subluxation of the carpal bones. This may ultimately lead to the degeneration of the radiocarpal joint, commonly known as Scapholunate Advanced Collapse (SLAC) wrist, which is a degenerative joint disease that progresses from the radiocarpal to the midcarpal joint line [2].

Early diagnosis and treatment are imperative to ensure a satisfying patient outcome. While wrist arthroscopy is considered the diagnostic gold standard, it is also a highly invasive procedure [8]. In 2021, the I-WRIST (International Wrist Radiologic evaluation for the Instability of the Scapholunate Joint) consortium, comprising hand surgeons and radiologists, issued a consensus statement for diagnostic imaging of SLJ instability. The panel recommended standard dorsopalmar and lateral wrist radiographs as well as stress views as part of the routine work-up. Additionally, dynamic fluoroscopy was proposed as an equally viable alternative method [9]. 

Radiographic widening of the scapholunate interval can be a sign of scapholunate instability [10]. However, in cases of isolated injury, the scapholunate interval may appear normal due to compensation by the secondary stabilizers mentioned above [11]. Occult scapholunate instability may become evident on radiographic stress views, such as the clenched ball view [2,9].

According to the I-Wrist panel, high-resolution ultrasonography may serve as a useful diagnostic addition for the examination of soft tissues with limited sensitivity [9,12,13]. Furthermore, MR-Arthrography was considered more sensitive to SLIL tears compared to standard MRI [14]. However, kinematic CT- or MR-studies were not recommended by the panel due to a lack of standardization [9].

Acute SLIL injury is typically treated surgically, with a range of surgical treatment options, among which dorsal capsulodesis is the most commonly employed. A recent meta-analysis has demonstrated that the established surgical treatment options are reliable strategies for decreasing pain and maintaining wrist function [15]. 

Real-time MRI (rtMRI) has emerged as a potent modality for capturing images at a high frame rate to visualize motion. In 2010, Uecker et al. introduced an innovative approach to real-time MRI, achieving a potential temporal resolution of 20 ms, which is equivalent to a frame rate of 50 per second. The technique was built on fast low angle shot (FLASH) acquisitions and undersampled radial k-space data combined with iterative image reconstruction. The radial acquisition pattern utilized involves filling k-space with spokes that traverse through the center, significantly enhancing the efficiency of the k-space sampling compared to the conventional line-by-line approach. The integration of iterative image reconstruction further optimizes the acquisition by allowing a reduction in the number of spokes, resulting in accelerated image acquisition. The flexibility to determine temporal resolution and image quality is achieved by adjusting acquisition parameters [16,17,18]. 

Recent studies by Krohn et al. have demonstrated the potential of this approach for the robust and efficient assessment of the temporomandibular joint with a temporal resolution of 66.7 ms [19]. Furthermore, Shaw et al. have demonstrated rtMRI of the wrist during radial and ulnar deviation as well as fist clench maneuvers to be feasible and useful for the evaluation of dynamic carpal instability [20].

In this study, our objective is to harness rtMRI for dynamic assessments of the scapholunate joint width (SLJW) in individuals without wrist pathology. Our hypothesis posits that rtMRI can provide accurate and reliable measurements of the SLJW during wrist motion. Through comparative analysis, we will determine the most reliable measurement technique and evaluate its inter- and intraobserver reliability. This study is designed as a feasibility study, serving as an initial step toward integrating rtMRI into standard wrist imaging protocols for the detection of carpal instability.

## 2. Materials and Methods

### 2.1. Study Design and Participants

We designed a prospective, mono-center feasibility study at the University Medical Center of the University of Göttingen, Germany. Eleven healthy participants without history of surgical intervention regarding the wrist or prior wrist injury were recruited for this study with the approval of the ethics committee. Exclusion criteria included any symptoms related to possible wrist injuries and pre-existing conditions related to the wrist joints, ligament diseases, or other rheumatologic conditions. Additionally, individuals with contraindications for MRI examination were excluded. Participants were instructed to refrain from engaging in any physical activities involving wrist strain for 48 h prior to the examination. 

### 2.2. Participant Preparation

Prior to the actual examination, participants received comprehensive training on the examination, which was explained in detail, and practiced outside the scanner. Special attention was given to clearly explain the required intensity, speed and range of motion.

Following the training, the participants were placed in the MRI scanner (3T Skyra, Siemens Healthineers, Erlangen, Germany) in a prone position. The right arm was extended forward, and the right hand was positioned on an examination pillow. A 16-channel multipurpose coil (Variety, Noras MRI products GmbH, Höchberg, Germany) was then positioned over the extended hand, ensuring sufficient space to perform the prescribed movements.

### 2.3. Image Acquisition

The imaging process consisted of both static and dynamic sequences, resulting in a total acquisition time of approximately 30 min. Initially, static T1-weighted Spin Echo images were acquired in the coronal and transverse planes before the dynamic sequence to identify potential occult pathologies within the examination area. These static images were used to define the region of interest (ROI) for the subsequent dynamic sequence. 

Dynamic imaging involved T1-weighted rtMRI sequences based on a RF-spoiled FLASH gradient-echo MRI technique (TR = 3.85 ms, TE = 2.28 ms, flip angle 4°). This technique was combined with a radial encoding scheme and iterative reconstruction by regularized nonlinear inversion (NLINV) [16,18]. 

**Figure 1:**
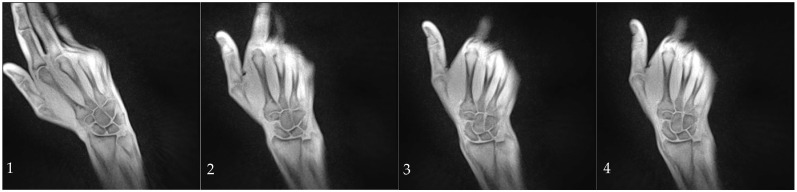
Series of images captured from radial (**1**) to ulnar deviation (**4**) during the abduction/adduction maneuver. These images were acquired using a T1-weighted rtMRI sequence with iterative reconstruction, achieving a temporal resolution of 50 ms. Images (**1**–**4**) correspond to frames 156, 207, 226, and 262 out of a total of 300 frames.

The participants were given commands through headphones to move radially or ulnarly within the previously practiced range of motion at a constant speed. The movement sequence was performed five times with two-minute breaks between each sequence. A subset of participants additionally performed a fist clench maneuver by compressing an elastic ball in a neutral position. During dynamic imaging, a series of 2D images were acquired every 50 milliseconds with an in-plane resolution of 0.75 mm and a slice thickness of 4 mm given a field of view of 192 mm and a matrix of 256 × 256 (Figure 1, Video S1 and S2). 

NLINV reconstruction of real-time images was carried out by a reconstruction computer (sysGen/TYAN Octuple-GPU, Sysgen; 8 GeForce GTX TITAN, Nvidia, Santa Clara, CA, USA) fully integrated into the commercial MRI system.

The acquired MRI data were post-processed using the software package MATLAB R2024a (MathWorks, Inc., Natick, MA, USA).

### 2.4. Measurement of the SL-Joint Space Width

**Figure 2:**
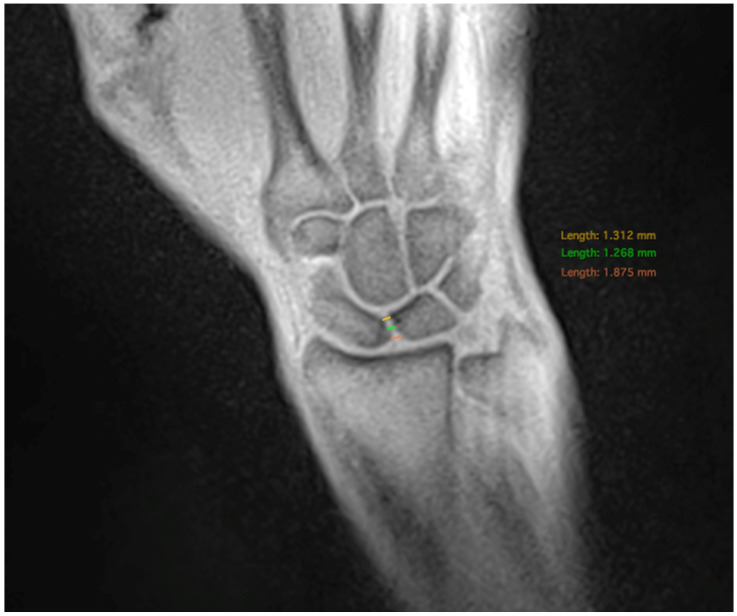
Measurement of the SLJW in three positions: at the proximal landmark (red), the medial landmark (green) and the distal landmark (yellow).

The SL distance was measured at three different positions with the distal, medial, and proximal bone points of the scaphoid and lunate serving as the anatomical landmarks. (Figure 2). Measurements were performed on both static and dynamic images. 

**Figure 3:**
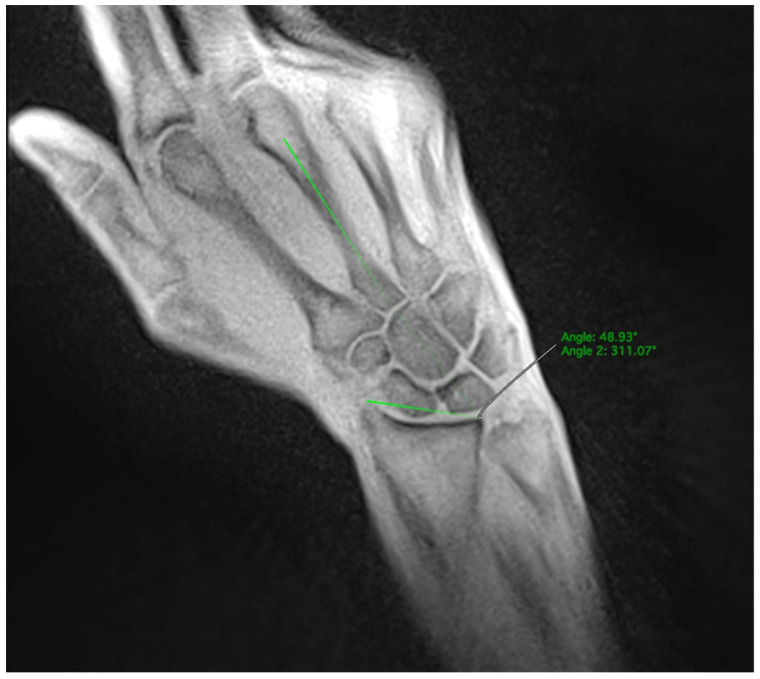
Measurement of the wrist angle determined by the extension of the third metacarpal and the distal radial joint line.

In the dynamic sequences, the SL width was determined every ten images. The extend and direction of movement in the radial or ulnar direction were determined by the angle formed between a line from the styloid process of the radius to the ulnar edge of the radius and the extension of the third metacarpal bone. The angle increased during ulnar movements and decreased during radial movements (Figure 3). The wrist angle remained constant during the fist clench maneuver and was therefore determined only once.

As this study only included healthy individuals, we hypothesized that the SL width would remain constant during the performed movements. Therefore, measurements on dynamic sequences were compared to measurements on the established static sequence, which served as a diagnostic reference.

The entire measurement process for both movement patterns was repeated after a few weeks to assess the intraobserver variability during the measurement procedure. After a few more weeks, another set of measurements for both movement patterns was conducted by a second observer, who independently performed the measurements to determine the interobserver variability. Observer 1 was a resident with two years of experience who had been trained on reading MRI scans. Observer 2 was a senior radiologist with more than 10 years of experience in musculoskeletal imaging. Both observers were blinded to each other’s measurements.

The analysis of the real-time MR images was conducted using the Software Horos (GNU Lesser General Public License, UK version 3.3.6, LGPL-3.0).

### 2.5. Statistical Analysis

First averages, ranges, maxima and minima were computed separately for each combination of participant, observer, maneuver, anatomical landmark, the two sets of measurements carried out by the first observer and additionally the direction of movement. Then, means and standard deviations were determined. 

Next, the average SL width on static images was determined. Mean absolute differences between measurements on static and dynamic images were calculated.

Linear mixed-effects regression models with a random intercept per participant were applied to analyze the effect of wrist position, movement direction and pattern as well as intra- and inter-rater reliability. Heteroskedasticity was modeled to allow for differing variances across anatomical landmarks. 

To achieve approximately normally distributed residuals, the measured ranges and absolute differences between measurements on static and dynamic images were logarithmically transformed before the analysis. Pairwise comparisons were conducted using the Tukey method with a significance level of 5%.

Analyses were performed in the statistical programming environment R (version 3.6.2; R Core Team 2019) using the packages nlme (version 3.1.153) and lmerTest (version 3.1.3) for linear mixed-effects regression models and emmeans (version 1.6.0) for post hoc testing.

## 3. Results

Initially eleven participants were recruited for the trial. However, one participant was excluded due to chronic degeneration of the radiocarpal joint incidentally found during static imaging. Ten participants performed the ab-/adduction maneuver, eight of which additionally performed the fist clench maneuver. Between the two types of motion, the SL width and the range showed no significant difference (*p* = 0.17 and *p* = 0.535). Determined by the above-mentioned wrist angle, the ab-/adduction maneuver was divided into abduction and adduction in order to analyze both movements separately. There was no significant difference between the two directions of movement regarding the SL width (*p* = 0.103) or range (estimated percentage difference 0.1, 95% CI −11–12.5, *p* = 0.99).

### 3.1. Distal Landmark

For the ab-/adduction maneuver, the average SL width was 2.1 mm (SD ± 0.2) at the distal landmark and 2.1 mm (SD ± 0.2) during the fist clench maneuver as well. The average range was 1.4 mm (SD ± 0.4) for the fist clench maneuver and 1.3 mm (SD ± 0.3) for ab-/adduction. The average SL width was 2.1 mm (SD ± 0.2) on both radial and ulnar movements with a mean range of 1.1 mm (SD ± 0.3).

### 3.2. Medial Landmark

For the ab-/adduction maneuver, the average SL width was 2.0 mm (SD ± 0.1) at the medial landmark. The average range was 1.1 mm (SD ± 0.6) for the fist clench maneuver and 0.85 mm (SD ± 0.17) for ab-/adduction. The average SL width was 2.1 mm (SD ± 0.2) on radial movements and 2.0 mm (SD 0.1) on ulnar movements. The mean range was 0.69 mm (SD ± 0.21) and 0.74 (SD ± 0.08), respectively.

### 3.3. Proximal Landmark

**Figure 4:**
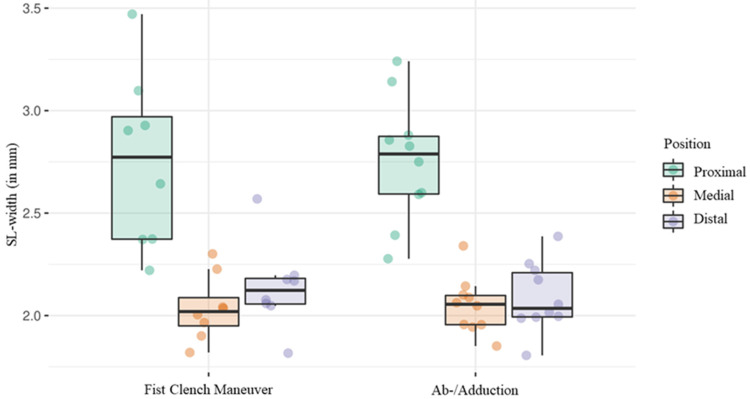
Boxplot diagram showing the average SL width during the fist clench and the ab-/adduction maneuver regarding each position separately.

For the ab-/adduction maneuver, the average SL width was 2.8 mm (SD ± 0.3) at the proximal landmark and 2.8 mm (SD ± 0.4) during the fist clench maneuver. Ranges averaged at 1.7 mm (SD ± 0.7) for the fist clench maneuver and 2.0 mm (SD ± 0.4) for ab-/adduction. The average SL width was 2.9 mm (SD ± 0.2) on radial movements and 2.7 mm (SD ± 0.3) on ulnar movements. The mean range was 1.6 mm (SD ± 0.4) and 1.6 mm (SD ± 0.5), respectively (Figure 4 and Table 1).

### 3.4. Comparison of the Three Landmarks

Measurements at the distal and medial anatomical landmark did not differ significantly (*p* = 0.091, Table 2). However, measurements at the distal and medial landmarks showed significant differences compared to the proximal landmarks considering all measurements as well as considering the ab/adduction and fist clench maneuver separately (all *p* < 0.001, Table 2).

Considering data from both maneuvers, substantial differences in ranges were observed at the three landmarks, with the medial range being 52.1% less than that at the proximal landmark, and the distal range exhibiting a 52.2% increase compared to the medial range (Table 3). 

### 3.5. Comparison with the Reference Standard

In the static images, the mean SL width was 2.7 mm (SD ± 0.3) at the proximal bone point, 2.2 mm (SD ± 0.1) at the medial landmark and 2.1 mm (SD ± 0.2) at the distal landmark. The smallest mean absolute deviation from the reference standard was measured at the medial landmark with 0.25 mm (SD ± 0.05) during the fist clench maneuver and 0.21 mm (SD ± 0.5) during the ab-/adduction maneuver. The average deviation showed no significant difference between the two performed movements (*p* = 0.311, Table 4). 

### 3.6. Intraobserver Variability

**Figure 5:**
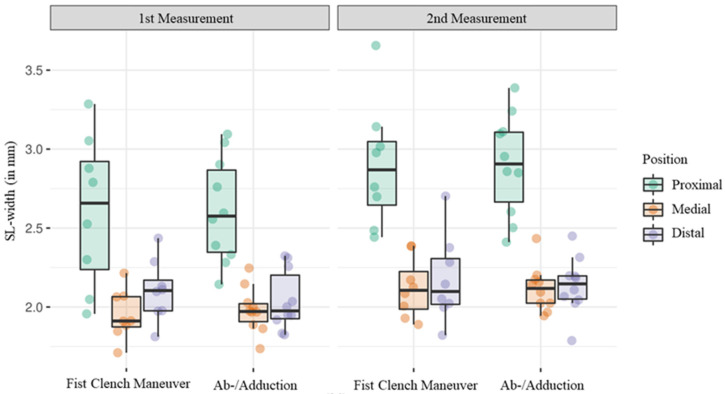
Boxplot diagram showing the average SL widths measured by observer 1 at two different points in time.

The average SL width during the first measurement by observer 1 was 2.2 mm (SD ± 0.2) with an average range of 1.2 (SD ± 0.3). During the second measurement, the average SL width was 2.4 mm (SD ± 0.2), with an average range of 1.6 mm (SD ± 0.4, Figure 5). There was a significant difference in the measurements of the SL width between the two timepoints (*p* < 0.001), being estimated as 0.14 mm (95% CI 0.09–0.20). The mean range was estimated to be increased by 33.4% (95% CI 19.9–48.5) in the second reading performed by observer 1. This observation was consistent for the individual maneuvers as well. Furthermore, linear mixed-effects regression models with maneuver and set of measurements as fixed effects were applied to the measurements obtained at the medial landmark only, revealing significant intraobserver variability as well (estimated difference 0.15, 95% CI 0.08–0.23, *p* < 0.001).

### 3.7. Interobserver Variability

**Figure 6:**
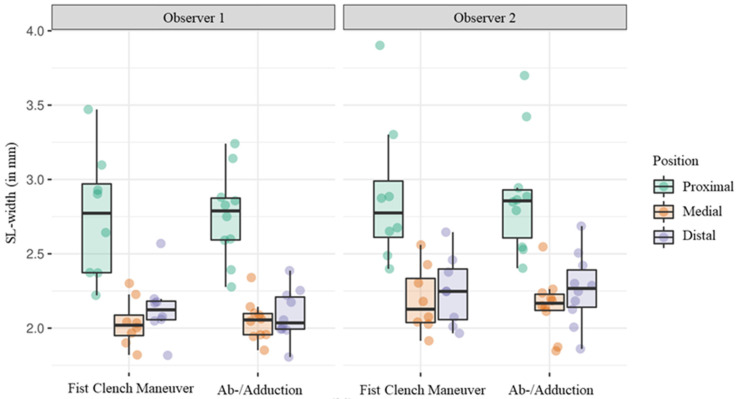
Boxplot diagram showing average SL-widths measured by observer 1 and 2.

To assess interobserver variability, the two measurements conducted by observer 1 were averaged. The mean SL width was determined to be 2.3 mm (SD ± 0.2) by observer 1 and 2.5 mm (SD ± 0.3) by observer 2 (Figure 6). The mean range was 1.4 mm (SD ± 0.3) for observer 1 and 1.6 mm (SD ± 0.3) for observer 2. Measurements differed significantly between the two observers when considering both maneuvers (estimated difference 0.14, 95% CI 0.08–0.19, *p* < 0.001) as well as when considering the fist clench maneuver (estimated difference 0.15, 95% CI 0.09–0.21, *p* < 0.001) and the ab/adduction maneuver (estimated difference 0.13, 95% CI 0.08–0.18, *p* < 0.001) separately. The difference between the observers did not differ significantly between the two maneuvers (*p* = 0.913). 

In relation to the mean absolute deviation from the reference standard, a notable difference was observed between the measurements of the two observers with observer 2 exhibiting an estimated 15.4% (95% CI 4.6–27.3, *p* = 0.005) increase in mean absolute deviation from the reference standard. 

Furthermore, linear mixed-effects regression models with maneuver and observer as fixed effects were applied to the measurements acquired at the medial landmark only, demonstrating significant interobserver variability as well (estimated difference 0.13, 95% CI 0.05–0.21, *p* = 0.003).

## 4. Discussion

In this study, we showcased the capability of rtMRI in visualizing the SL joint during wrist motion. To date, dynamic wrist examination has predominantly relied on fluoroscopy, resulting in radiation exposure for both the patient and the examiner [10]. Furthermore, fluoroscopy offers only indirect information about the complex anatomy of the wrist as only osseous structures are displayed, leaving soft tissue mostly invisible. In clinical practice, stress views such as the clenched-ball view can be obtained using plain radiographs [9]. However, both fluoroscopy and stress views yield summation images, which can make precise measurements of the scapholunate interval difficult.

Ultrasonography can be a helpful tool in the hands of an experienced examiner and offers great soft tissue contrast. However, the technique is heavily dependent on the experience level of the examiner. Dao et al. reported a sensitivity of 46.2% for ultrasound in the detection of carpal instability and a specificity of 100% [13]. The initially mentioned I-WRIST panel did not recommend ultrasonography to be a part of a routine work-up [9].

Static MRI or MRA are both recommended by the panel. A study by Kader et al. reported a specificity of 90.5% and 91% respectively. Sensitivity was 57.7% for MRA and only 19.2% for standard MRI [9,14]. 

Presently, clinicians are faced with a dilemma, having to balance the trade-off between soft tissue contrast and kinematic information. Previous studies have demonstrated the potential of rtMRI [19]. For instance, in the domain of cardiac imaging, rtMRI sequences have emerged as a potent tool for anatomical and functional studies [21]. The application of this technique in wrist imaging holds significant promise, as it capitalizes on the advantages of MRI and dynamic imaging.

This study demonstrates the high accuracy of the SL width measurements in healthy individuals using rtMRI compared to established static T1-weighted images with average absolute deviations falling within the submillimeter range. However, among the three anatomical landmarks, the medial landmark exhibited superior results, displaying less deviation from the reference standard and less scatter compared to the proximal and distal landmark. This finding is unexpected, considering that the SLIL primarily connects the proximal bone poles, leading to the anticipation of more stable measurements observed at the proximal landmark [1,2]. However, the translational movement experienced by the proximal bone poles during the maneuvers may have influenced the results. Nevertheless, consistent results were observed during the fist-clench maneuver, which did not involve any translational movement. 

As previously indicated, kinematic studies have revealed that despite the ab/adduction maneuver being executed in a single plane, the scaphoid and lunate bones undergo a degree of flexion during radial deviation and extension during ulnar deviation [6]. Given the complexities introduced by this three-dimensional movement, we propose future approaches should integrate three-dimensional image acquisition to address partial volume effects arising from the intricate shapes of carpal bones.

On plain radiography and fluoroscopy, a SLJW of up to 2 mm is considered normal, while values exceeding 3 mm are indicative of an SL lesion [22,23]. For dynamic fluoroscopy, a sensitivity of 90% and a specificity of 97% has been reported for the detection of carpal instability [24]. In our study, measurements at the medial bone points closely approximated the joint width considered normal on plain radiography and dynamic fluoroscopy, suggesting that future research might consider measurements at the medial landmark to be the most accurate. Changes of SLJW at the medial landmark may be particularly sensitive to pathological widening.

Our findings suggest that the fist clench maneuver is similarly suited for real-time MRI (rtMRI) measurements compared to the ab/adduction maneuver. However, it is important to acknowledge that the physiological dorsal extension of the wrist, mediated by the Mm. extensor carpi radialis longus et brevis during the ab/adduction maneuver, might lead to an angulation of the carpal bones. This angulation could potentially compromise the alignment with the coronally oriented rtMRI sequence. Therefore, the dynamic acquisition during the ab/adduction maneuver which is performed in the same plane as the coronally oriented rtMRI sequence, might after all still be a superior choice in terms of reproducibility. 

Inter- and intraobserver comparisons revealed notable variability, emphasizing the need for further improvement and standardization of the technique. As measurements at the medial landmark provided the most promising results, we conducted an additional distinct intra- and interobserver comparison. However, we observed significant inter- and intraobserver variability, potentially stemming from edge blurring, leading to inconsistent measurements. Future advancements in Deep Learning (DL) may enhance image quality and contour delineation [25]. Additionally, automated, DL-based post-processing could offer a more standardized approach with improved reproducibility [26,27].

The FLASH sequence utilized in this study generated T1-weighted images with a temporal resolution of 20 ms, which is equivalent a frame rate of 50 fps. Mazzoli et al. successfully employed a bSSFP sequence in order to acquire fat suppressed images of the knee and the wrist at 3T. The acquisition showed good fat–water separation at the expense of the temporal resolution, which was 10 fps for the wrist [28]. Consequently, FLASH-based rtMRI holds the potential to nearly double the temporal resolution.

This study has several limitations.

We initially assumed complete stiffness of the SLIL. However, biomechanical studies have demonstrated that this assumption does not align with reality [4,5]. Nevertheless, we do not anticipate a significant impact on the results due to the overall low elasticity of the SLIL. 

The measurements were conducted manually in a time-consuming process and may be susceptible to errors. 

The results regarding intra- and interobserver variability should be interpreted with caution, since they are based on only one and two observers, respectively.

Additionally, all measurements were rounded to the third decimal, and the statistical results were rounded to the first decimal. This might falsely imply an accuracy that exceeds the in-plane resolution achieved with the parameters used. Few images showed artifacts from unintended wrist movements outside of the trained procedure. This issue could potentially be addressed in the future through the development of a specifically designed orthosis.

Also, this study only included healthy individuals. The diagnostic value of rtMRI for the diagnosis of carpal instability will have to evaluated in future trials including patients with known wrist pathology.

In conclusion, rtMRI exhibits promise as a technique for dynamic examination of the SL joint with high temporal resolution. The measurements of the SL joint width demonstrate strong agreement with established MRI methods. However, further standardization and improvement of the technique may be necessary to achieve sufficient intra- and interobserver agreement. The findings of this study suggest that measurements of the SL width are most robust at the medial landmark.

## Figures and Tables

**Table 1:** Average SL width, range, minimum and maximum during the fist clench and the ab-/adduction maneuver regarding each position separately.

		Proximal	Medial	Distal
Ab-/Adduction	Mean average SL-width in mm (SD)	2.8 (±0.4)	2.0 (±0.2)	2.1 (±0.2)
	Mean range in mm (SD)	2.0 (±0.4)	0.9 (±0.2)	1.3 (±0.3)
	Mean minimum in mm (SD)	1.9 (±0.2)	1.6 (±0.1)	1.5 (±0.2)
	Mean maximum in mm (SD)	3.9 (±0.5)	2.5 (±0.2)	2.9 (±0.3)
Fist Clench Maneuver	Mean average SL-width in mm (SD)	2.8 (±0.3)	2.0 (±0.1)	2.1 (±0.2)
	Mean range in mm (SD)	1.7 (±0.7)	1.1 (±0.6)	1.4 (±0.4)
	Mean minimum in mm (SD)	2.0 (±0.4)	1.6 (±0.2)	1.6 (±0.1)
	Mean maximum in mm (SD)	3.8 (±0.8)	2.7 (±0.7)	2.9 (±0.5)

**Table 2:** Comparison of average SL width across the ab-/adduction and fist clench maneuver as well as the three measurement positions using linear mixed-effects models with post hoc Tukey tests. Only measurements from the first observer were considered. All models included the set of measurements (first or second) as fixed effect.

	Comparison of Average SL-Width		
	Estimated Difference	95% CI	*p*-Value
**Both maneuvers**			
Medial vs. Proximal	−0.71	(−0.84, −0.58)	<0.001
Distal vs. Proximal	−0.64	(−0.78, −0.5)	<0.001
Distal vs. Medial	0.07	(−0.01, 0.14)	0.091
Ab-/adduction vs. Fist clench	−0.041	(−0.1, 0.018)	0.170
**Fist clench maneuver**			
Medial vs. Proximal	−0.71	(−0.93, −0.5)	<0.001
Distal vs. Proximal	−0.61	(−0.84, −0.38)	<0.001
Distal vs. Medial	0.1	(−0.02, 0.22)	0.106
**Ab-/Adduction maneuver**			
Medial vs. Proximal	−0.74	(−0.85, −0.63)	<0.001
Distal vs. Proximal	−0.71	(−0.83, −0.59)	<0.001
Distal vs. Medial	0.03	(−0.04, 0.1)	0.594
Ulnar vs. Radial	−0.04	(−0.09, 0.01)	0.103

**Table 3:** Comparison of mean ranges measured across the three different positions using linear mixed-effects models with post hoc Tukey tests. Only measurements from the first observer were considered. The model included the set of measurements (first or second) as fixed effect.

	Comparison of Mean Ranges		
	Estimated Percentage Difference	95% CI	*p*-Value
Medial vs. Proximal	−52.1	(−59.6, −43.2)	<0.001
Distal vs. Proximal	−27.1	(−36.9, −15.7)	<0.001
Distal vs. Medial	52.2	(27, 82.4)	<0.001
Ab-/adduction vs. Fist clench	3.6	(−7.4, 15.8)	0.535

**Table 4:** Comparison of absolute differences from the reference standard across the different maneuvers and positions using linear mixed-effects models with post hoc Tukey tests. The two measurements from the first observer were averaged. The model included the observer as fixed effect.

Absolute Difference from Reference			
	Estimated Percentage Difference	95% CI	*p*-Value
**Both maneuvers**			
Medial vs. Proximal	−51.2	(−59.6, −41.1)	<0.001
Distal vs. Proximal	−40.4	(−50.8, −27.8)	<0.001
Distal vs. Medial	22.2	(7.7, 38.6)	0.001
Ab-/adduction vs. Fist clench	−5.1	(−14.3, 5.1)	0.311
**Fist-clench maneuver**			
Medial vs. Proximal	−46	(−58.4, −30)	<0.001
Distal vs. Proximal	−41.3	(−54.5, −24.4)	<0.001
Distal vs. Medial	8.6	(−9.6, 30.5)	0.519
**Ab-/Adduction**			
Medial vs. Proximal	−55	(−65.6, −41.3)	<0.001
Distal vs. Proximal	−39.7	(−53.5, −21.7)	<0.001
Distal vs. Medial	34.2	(15.3, 56.3)	<0.001

## Data Availability

The data presented in this study are available in this article.

## Supplementary Materials

The following supporting information can be downloaded at https://www.mdpi.com/article/10.3390/diagnostics14111177/s1, Video S1: ab/adduction_movie, Video S2: fist_clench_movie.
